# How Machine Learning is Powering Neuroimaging to Improve Brain Health

**DOI:** 10.1007/s12021-022-09572-9

**Published:** 2022-03-28

**Authors:** Nalini M. Singh, Jordan B. Harrod, Sandya Subramanian, Mitchell Robinson, Ken Chang, Suheyla Cetin-Karayumak, Adrian Vasile Dalca, Simon Eickhoff, Michael Fox, Loraine Franke, Polina Golland, Daniel Haehn, Juan Eugenio Iglesias, Lauren J. O’Donnell, Yangming Ou, Yogesh Rathi, Shan H. Siddiqi, Haoqi Sun, M. Brandon Westover, Susan Whitfield-Gabrieli, Randy L. Gollub

**Affiliations:** 1 Department of Psychiatry and Martinos Center for Biomedical Imaging, Department of Radiology, Massachusetts General Hospital, Boston, MA 02114, USA; 2 Harvard-MIT Health Sciences and Technology, Massachusetts Institute of Technology, Cambridge, MA 02139, USA; 3 University of Massachusetts Boston, Boston, MA 02125, USA; 4 Department of Radiology, Boston Children’s Hospital, Harvard Medical School, Boston, MA 02115, USA; 5 Department of Neurology and McCance Center for Brain Health / Harvard Medical School, Massachusetts General Hospital, Boston 02114, USA; 6 Centre for Medical Image Computing, University College London, London, UK; 7 Martinos Center for Biomedical Imaging, Department of Radiology, Massachusetts General Hospital and Harvard Medical School, Boston 02114, USA; 8 Computer Science and Artificial Intelligence Laboratory, Massachusetts Institute of Technology, Cambridge, MA 02139, USA; 9 Institute of Systems Neuroscience, Medical Faculty, Heinrich Heine University Düsseldorf, Düsseldorf, Germany; 10 Institute of Neuroscience and Medicine, Brain & Behaviour (INM-7) Research Centre Jülich, Jülich, Germany; 11 Department of Radiology, Brigham and Women’s Hospital and Harvard Medical School, MA 02115 Boston, USA; 12 Department of Psychology, Northeastern University, Boston 02115, USA; 13 Martinos, Radiology, MGH, MIT, HMS & EECS, Cambridge 02114, USA; 14 Department of Psychiatry, Brigham and Women’s Hospital and Harvard Medical School, Boston 02115, USA; 15 Center for Brain Circuit Therapeutics, Department of Neurology, Psychiatry, and Radiology, Brigham and Women’s Hospital and Harvard Medical School, 02115 Boston, USA

**Keywords:** Machine learning, Deep learning, Clinical translational neuroimaging, Brain health, MRI, PET, EEG, Transcranial magnetic stimulation

## Abstract

This report presents an overview of how machine learning is rapidly advancing clinical translational imaging in ways that will aid in the early detection, prediction, and treatment of diseases that threaten brain health. Towards this goal, we aresharing the information presented at a symposium, “Neuroimaging Indicators of Brain Structure and Function - Closing the Gap Between Research and Clinical Application”, co-hosted by the McCance Center for Brain Health at Mass General Hospital and the MIT HST Neuroimaging Training Program on February 12, 2021. The symposium focused on the potential for machine learning approaches, applied to increasingly large-scale neuroimaging datasets, to transform healthcare delivery and change the trajectory of brain health by addressing brain care earlier in the lifespan. While not exhaustive, this overview uniquely addresses many of the technical challenges from image formation, to analysis and visualization, to synthesis and incorporation into the clinical workflow. Some of the ethical challenges inherent to this work are also explored, as are some of the regulatory requirements for implementation. We seek to educate, motivate, and inspire graduate students, postdoctoral fellows, and early career investigators to contribute to a future where neuroimaging meaningfully contributes to the maintenance of brain health.

## Introduction

Machine learning is contributing to rapid advances in clinical translational imaging to enable early detection, prediction, and treatment of diseases that threaten brain health. Brain diseases, including cerebrovascular disease, depression, migraine headaches, and dementia, are leading causes of global disability (Vos et al., 2020). Continued progress in neuroimaging and machine learning, and the collection of increasingly large-scale data sets, promise to transform healthcare by providing non-invasive, reliable indicators of brain health, resilience, and vulnerability long before clinical manifestations of disease. But many technical challenges remain. On February 12th 2021, the MGH McCance Center for Brain Health at Mass General Hospital, together with the Harvard-MIT Health Sciences and Technology Neuroimaging Training Program, co-hosted a virtual symposium, “Neuroimaging Indicators of Brain Structure and Function—Closing the Gap Between Research and Clinical Application,” to highlight some of these remaining challenges and machine learning approaches to overcome them. Recorded videos of the symposium presentations and discussions are available link to YouTube videos https://www.youtube.com/playlist?list=PL0A-NKHLVrNF82vdjeyyaBRoiXg77lCeW.

**Fig. 1:**
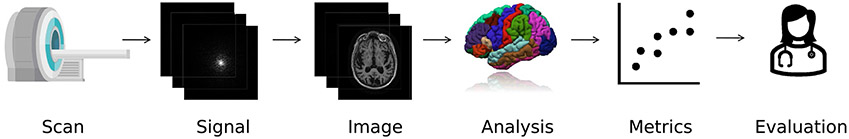
Schematic illustration of the spectrum of machine learning applications in clinical translational neuroimaging. A typical volumetric neuroimaging workflow is shown for MRI. A patient is scanned, creating a signal (i.e. k-space data) which is converted to an image via a reconstruction algorithm in preparation for clinical review by a radiologist. In some cases, the reconstructed image undergoes further computational processing to produce higher-level summaries, such as segmentations or registrations to an atlas. Optionally, in the future, further computational processing can convert these summaries to metrics used to quantify brain health, such as the volume of a structure or the estimated age of a subject. Finally, these quantitative metrics, once comprehensively validated, can be used to inform patient care through early detection of subtle abnormalities and to guide treatments such as targeted brain stimulation. These steps are not just useful at the individual patient level but can also drive population level analyses that can lead to insights regarding healthy and disordered brain structure and function

In this symposium report, we explore a spectrum of machine learning applications in neuroimaging and use symposium presentations to illustrate key points. We cover both recent advances and outstanding challenges, beginning with image acquisition, and ending with computation of quantitative metrics and initial clinical utilization (Fig. 1):

**Section I** describes how machine learning improves volumetric image acquisition and reconstruction.

**Section II** describes machine learning approaches to image processing, focusing on image harmonization, and methods to detect deviations from healthy brain structure and function.

**Section III** describes machine learning advances in interpretation and analysis of non-volumetric EEG data.

**Section IV** describes multiple approaches to assess brain health using deviations from healthy aging.

**Section V** describes how machine learning techniques can be applied to individual patient imaging, and other diagnostics, to personalize medical treatments that improve brain health.

Finally, **Section VI** explores the implications of deploying neuroimaging indicators of brain health into a clinical workflow. In particular, we focus on regulatory approval pathways of machine learning algorithms and the ethical considerations involved in collecting, algorithmically analyzing, and acting upon the derived information. We raise a set of important questions that we believe researchers should bear in mind when working in this area.

Machine learning models vary in the amount of domain knowledge they incorporate and how they do so. Some models explicitly enforce that their outputs are consistent with the physics of an imaging or measurement process. Other methods act upon features explicitly chosen because they are known to be relevant for the task at hand. These feature-based methods are often applied to established clinical use cases to reduce time, manual labor, and/or person-to-person variation (Gajawelli et al., 2019). "End-to-end" approaches abstract out explicit feature definition to go from raw data all the way to interpretable quantitative metrics, for example, of brain health. All traditional step-by-step processes, such as artifact removal, registration, conversion between temporal and spectral domains, and feature extraction could be encompassed in a single machine learning pipeline. In general, models with more explicitly encoded domain knowledge are less flexible in adapting to cases where the measurement process may be inaccurately characterized. That said, these methods are able to incorporate known relationships, which can guide the learning process and prevent nonsensical results. The methods covered in this report represent examples from each of these categories as well as intermediate cases which explicitly incorporate some domain knowledge but also allow the model significant flexibility in producing unconstrained final outputs.

It is important to acknowledge that the field is dynamic with each area undergoing rapid transitions. In some cases, machine learning techniques are already deployed or in advanced stages of testing for deployment into existing clinical workflows. In other cases, efforts are focused on early research, with the goal of discovering scientific insight or extracting meaningful features from the images that can be fed into machine learning methods to generate clinically meaningful biomarkers (FDA-NIH Biomarker Working Group, 2016; Mateos-Pérez et al., 2018). In all presentations and throughout this report, our goal is to educate, motivate and inspire graduate students, post-doctoral fellows, and early career investigators to contribute to a future where imaging meaningfully contributes to the maintenance of brain health.

## Section I: Machine Learning for Improved Volumetric Image Acquisition and Reconstruction

Machine learning techniques can be used to improve one of the earliest steps in the neuroimaging pipeline even before an image is viewed by a clinician or researcher: image formation. Typically, scanner-acquired measurements represent an encoding of the patient anatomy under the physics governing the imaging system. For example, the measurements acquired from an MRI scanner represent the Fourier transform of the image of interest (Nishimura, 2010), while the measurements acquired from PET and CT scanners represent the Radon transform of the image of interest (Ramm & Katsevich, 1996). Recovering the underlying image from the acquired scanner data requires solving an inverse problem. Due to time, patient comfort and safety considerations, or monetary constraints, often only a limited number of scanner measurements are acquired, making this inverse problem highly under-determined. Further, the acquired signals may be corrupted by imperfections in the imaging process, such as patient motion or system noise. Techniques from machine learning, including (1) model-based optimization methods (Griswold et al., 2002; Lustig et al., 2008; Pruessmann et al., 1999), (2) data-driven learning methods (Quan et al., 2018; Yang et al., 2018), and (3) combinations of these two strategies (Hammernik et al., 2018; Schlemper et al., 2018), are promising approaches for mitigating these image formation issues, enabling faster, higher-quality image creation for downstream analysis.

One such application of machine learning involves deciding which exact scanner measurements to acquire. Given time or financial imaging budget constraints, machine learning can be used to identify which subset of measurements will be the most informative for reconstructing the final image. For example, several approaches have been proposed for learning the optimal k-space acquisition pattern for a specified class of MRI scans, often identifying different patterns for different anatomies (Bahadir et al., 2020; Wang et al., 2021; Weiss et al., 2021). For neuroimaging applications in particular, sampling trajectories could be optimized for specific structures of interest for the clinical question being asked. More recent work aims to optimize the acquisition pattern with even greater specificity for each individual patient (Zhang et al., 2019).

Once all scanner data is acquired, machine learning is useful in reconstructing the image of interest itself. Model-based optimization techniques for MRI, CT, and PET imaging have typically provided iteratively refined solutions to under-determined inverse problems (Griswold et al., 2002; Lustig et al., 2008; Pruessmann et al., 1999). Recently, deep-learning methods quickly estimate solutions to the inverse imaging problems, including network architectures that explicitly employ the physics of the imaging system (Hammernik et al., 2018; Putzky et al., 2019; Schlemper et al., 2018). Efforts to collect large-scale public datasets of raw imaging data have accelerated advances in image reconstruction by enabling rapid model prototyping and by simplifying and standardizing evaluation of varying approaches. For example, the FastMRI dataset provides publicly available k-space data for reconstructing over six thousand human brain MRIs (Zbontar et al., 2019).

**Fig. 2:**
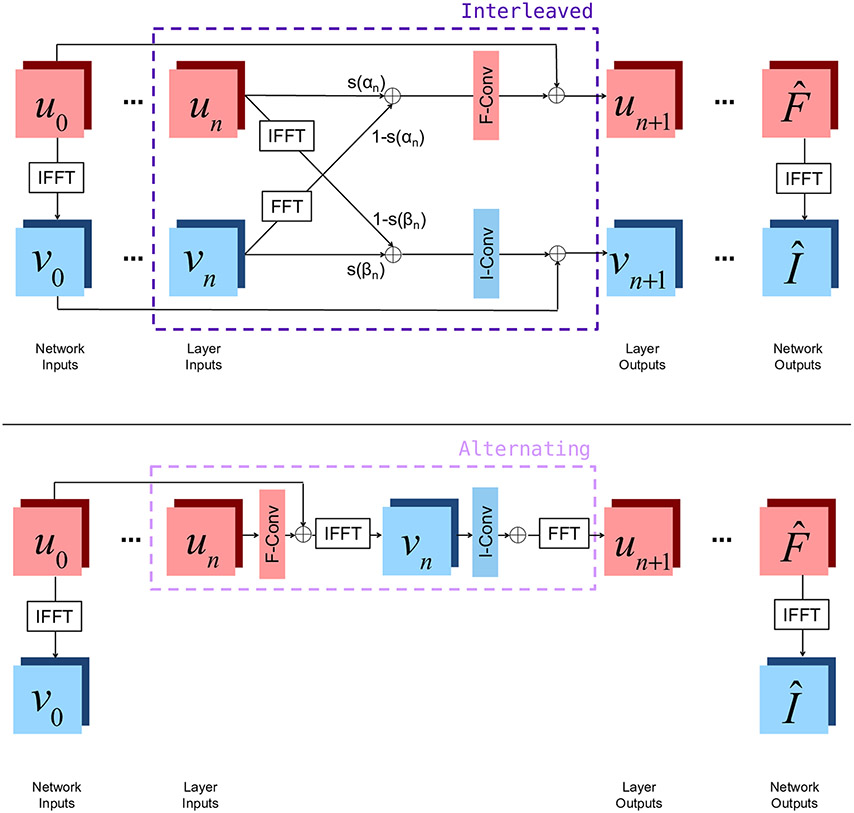
Two joint layer architectures combining frequency and image space representations, embedded within full network architectures for MRI reconstruction. Red squares represent frequency space quantities, while blue squares represent image space quantities. u_n_ represents frequency space features at the nth layer, and v_n_ represents image space features at the nth layer. At each layer, Batch Normalization (BN), a convolution, and an activation function are applied to both u_n_ and v_n_, summarized by ‘F-Conv' or ‘I-Conv', respectively

**Fig. 3:**
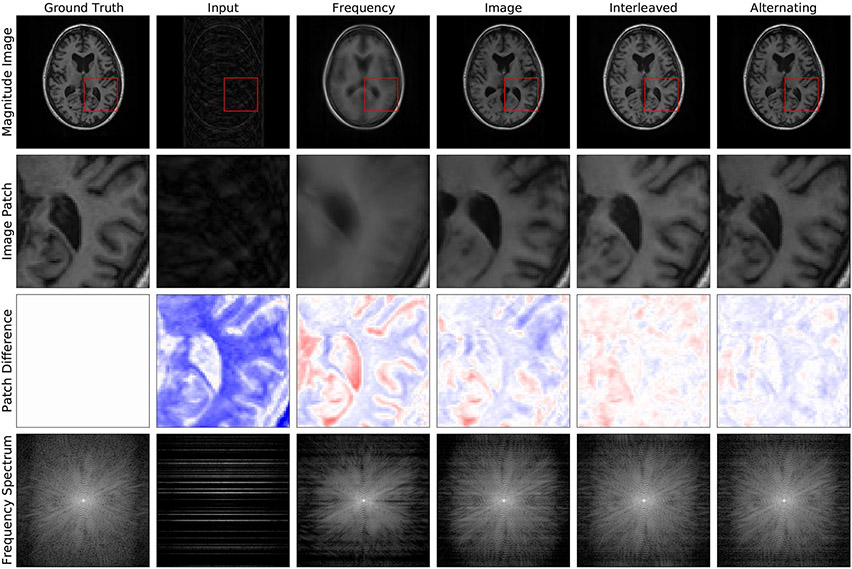
Example reconstructions from 4 × undersampled data (row 1), zoomed-in image patches (row 2), difference patches between reconstructions and ground truth images (row 3), and frequency space reconstructions (row 4) are shown here to visually communicate the impact of this reconstruction approach. It is most easily appreciated by comparing the final two columns. The Interleaved and Alternating architectures produce two slightly different reconstructions, both of which better eliminate blurring and 'ringing' artifacts, where multiple copies of the image appear stamped on top of each other

Beyond accelerated reconstruction from limited measurements, several machine learning approaches have been proposed for correcting artifacts arising during image acquisition and reconstruction. For example, both optimization and learning-based approaches have been proposed for MRI denoising (Anand & Sahambi, 2010; Manjon & Coupe, 2019) and motion correction (Chun et al., 2012; Haskell et al., 2018; Pipe, 1999). At this symposium, Ms. Nalini Singh presented two neural network layer structures which can be used to build networks which correct each of these artifacts while also being used for accelerated reconstruction (Singh et al., 2020). Unlike many other reconstruction methods, these layers incorporate convolutions on both the frequency space and image space features. By operating in both spaces, these layers both correct artifacts native to the frequency space and manipulate image space representations to form coherent image structures. Figure 2 shows a detailed diagram of the layers, and Fig. 3 shows example reconstructions demonstrating the positive impact of this method on the quality of the reconstructed image in representing the true brain anatomy.

Several deep-learning based approaches have also been proposed for metal artifact reduction in CT imaging of other anatomies (Gjesteby et al., 2017; Hu et al., 2019); these techniques could be extended to improve brain CT imaging for patients with deep brain stimulation (DBS) devices in situ. In PET imaging, deep-learning based approaches have demonstrated improved correction of attenuation effects both with (Ladefoged et al., 2018; Liu et al., 2018a) and without (Dong et al., 2020; Liu et al., 2018b) concordant anatomical imaging, or to enable low-dose PET (Xu et al., 2017). Each of these innovations makes critical contributions to improving the safety, quality and/or value of clinically meaningful information about brain health which can be gleaned from the imaging study.

While machine learning-based approaches promise to improve the speed, value, and quality of brain image acquisition, several challenges must be solved before they are incorporated into standard clinical workflows. For example, many current reconstruction methods require large datasets of thousands of high-quality acquired signals from a particular imaging protocol. This requirement makes it difficult to apply these methods to new imaging protocols for which large datasets have not yet been collected. New techniques are being developed to adapt these learning-based approaches to either require fewer training examples or to transfer the information from previously collected datasets of one protocol to a new protocol of interest (Han et al., 2018). And, for any reconstruction method, uncertainty quantification techniques will be needed to highlight regions of reconstructions with a high likelihood of error (Edupuganti et al., 2021). These uncertainty quantifications will enable radiologists to understand when more detail is needed to identify a particular feature of interest (Edupuganti et al., 2021), possibly requiring re-imaging of the patient.

## Section II: Machine Learning Applications for Volumetric Image Processing

Once brain imaging data is collected and reconstructed, there are several steps in the image analysis pipeline where machine learning can improve the extraction of meaningful, quantitative features related to brain health. In this section we explore some of these advances, giving an overview of each step and examples of how machine learning models are used. We focus on MRI to demonstrate how ML techniques can be incorporated in each workflow step, but similar principles extend to other volumetric imaging techniques such as PET and CT.

### Quality Assurance and Harmonization

Expert human labellers typically perform image quality assessment (QA), but this process is labor-intensive and can suffer from low inter-rater reliability. Carefully designed machine learning techniques promise to enable fast, easily accessible, consistent QA. Previously proposed approaches use carefully curated quality metrics as input features to various types of classifiers which label images as usable or unusable (Esteban et al., 2017; Küstner et al., 2018; Pizarro et al., 2016). Crowdsourcing approaches have also been used to improve the accuracy of these automatic QA tools. The use of many non-expert, human raters as inputs to a convolutional neural net improves the accuracy of classification over a single site data set (Keshavan et al., 2019). Further, a web-based API acting as a quality metric repository has increased the volume of quality metric labeled, multi-site data available to be used to develop new, more generalizable QA tools (Esteban et al., 2019).

In addition to ensuring the quality of individual scans, batch effects affecting images acquired at different locations or times must be eliminated to perform large-scale, multisite studies. Machine learning approaches provide flexible methods to detect and remove the relevant site-specific effects. One approach is to directly convert data acquired in one setting to the data that would have been acquired in a different setting. During the symposium, Dr. Cetin-Karayumak presented such a retrospective harmonization technique which represents diffusion MRI (dMRI) data as a combination of spherical harmonic basis functions. Rotationinvariant features are derived at each voxel from the computed basis function coefficients for each image, and a mapping is computed between the features of target and reference scanners in order to harmonize them (Cetin Karayumak et al., 2019). A different approach is to learn intermediate representations invariant to the scanner on which any image was acquired. These intermediate representations can then be used to reconstruct images without site-specific effects (Moyer et al., 2020). Alternatively, instead of removing or transforming site-specific effects at the image level, a third strategy is to encourage downstream features derived from the images for a machine learning prediction task to be invariant to the scanners on which they were acquired (Dinsdale et al., 2021).

### Quantification of Brain Health and Detection of Abnormality

**Fig. 4:**
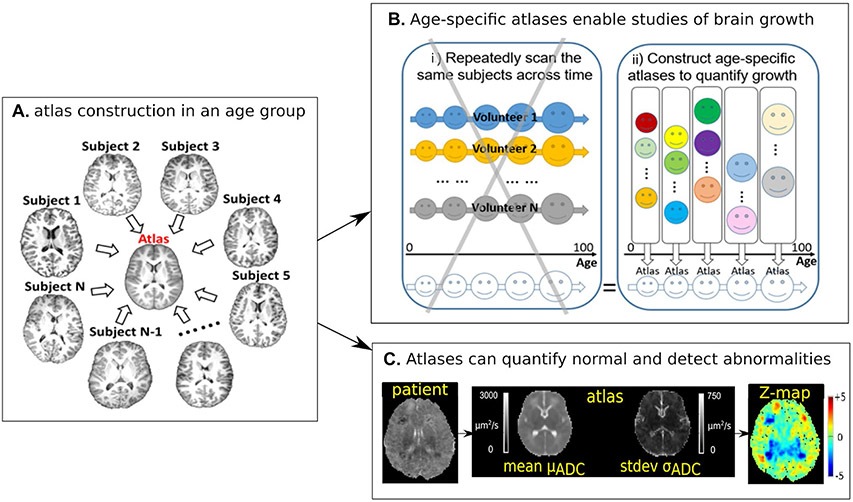
Atlas construction (concept in panel **A**) can enable quantification of brain development across ages (panel **B**- schematically indicating the benefits of using a clinical cohort of individuals for atlas construction versus a prospectively gathered longitudinal cohort) and can detect abnormalities as outliers to normal (panel **C**). ADC-Apparent Diffusion Coefficient

Machine learning approaches can also be used to characterize healthy brain characteristics and identify deviations from the norm. During the symposium, Dr. Yangming Ou described how to construct ‘normal’ atlases using groupwise unbiased image registration. Brain MRI atlases summarize healthy brain anatomy and typical signal intensity profiles at the voxel-, regional-, fiber-, and whole-brain levels (Guimond et al., 2000) (Fig. 4A). Brain atlases constructed from imaging data can be used in multiple ways to quantify brain health. One example is the quantification of normal childhood development (Ou et al., 2017; Sotardi et al., 2021). A series of constructed atlases from cohorts of healthy subjects clustered by age can enable longitudinal quantification of brain development from data sets where every subject was scanned once (Fig. 4B). This is not only cost effective when constructed from clinically acquired brain scans, but also has the potential to incorporate a more comprehensive range of healthy variation than data acquired in a single or set of pooled research studies. Another use of quantitative brain atlases is to detect subtle abnormalities due to a wide range of disorders (Pinto et al., 2018) (Fig. 4C). Atlas-quantified voxel-wise deviation values can be used as features in classical machine classifiers (O’Muircheartaigh et al., 2020) or deep convolutional neural networks (Baur et al., 2021) to further improve the accuracy and generality of atlas-based detection of deviations from brain health. This strategy has been used for structural MRI (Baur et al., 2021; O’Muircheartaigh et al., 2020) and diffusion MRI (Pinto et al., 2018).

### Segmentation

Automatic segmentation of brain images enables quantitative estimation of the volumes of brain structures that can lead to other indicators of brain health. These quantitative estimates enable population studies as well as longitudinal analysis within individual subjects. Most previous work on brain segmentation has focused on MRI, which provides detailed images with an ever increasing range of specifically tuned contrasts for visualizing different details of brain anatomy and function (Akkus et al., 2017).

Freesurfer is one example of a widely used package for brain MRI analysis and includes a machine learning approach to segment many brain structures (Fischl et al., 2002) as part of a larger image analysis pipeline. This technique involves finding the maximum a posteriori estimate of a segmentation of an anatomical brain region (e.g. hippocampus), given the image to be segmented and a linear transform mapping it to an expertly curated segmentation atlas. This technique only employs approximately one hundred labeled scans for a specific atlas, but the entire segmentation procedure takes several hours. There has therefore been recent interest in neural network-based segmentation methods, which provide segmentations on the order of seconds (Akkus et al., 2017; Despotović et al., 2015) to address the requirement to perform expert level segmentation on large scale image data sets.

In these approaches, a convolutional neural network typically directly predicts human-labeled segmentations from patches or volumes and requires many labeled images to train. Furthermore, these approaches are extremely sensitive to shifts in input image intensity. To apply these methods to scans of a different contrast or resolution, additional labels must be collected and used to retrain or fine-tune the networks. Thus, recent research has focused on unsupervised deep learning approaches for training brain segmentation networks, (Dalca et al., 2019) or on adapting trained networks to new imaging analysis task scenarios (Kamnitsas et al., 2016).

**Fig. 5:**
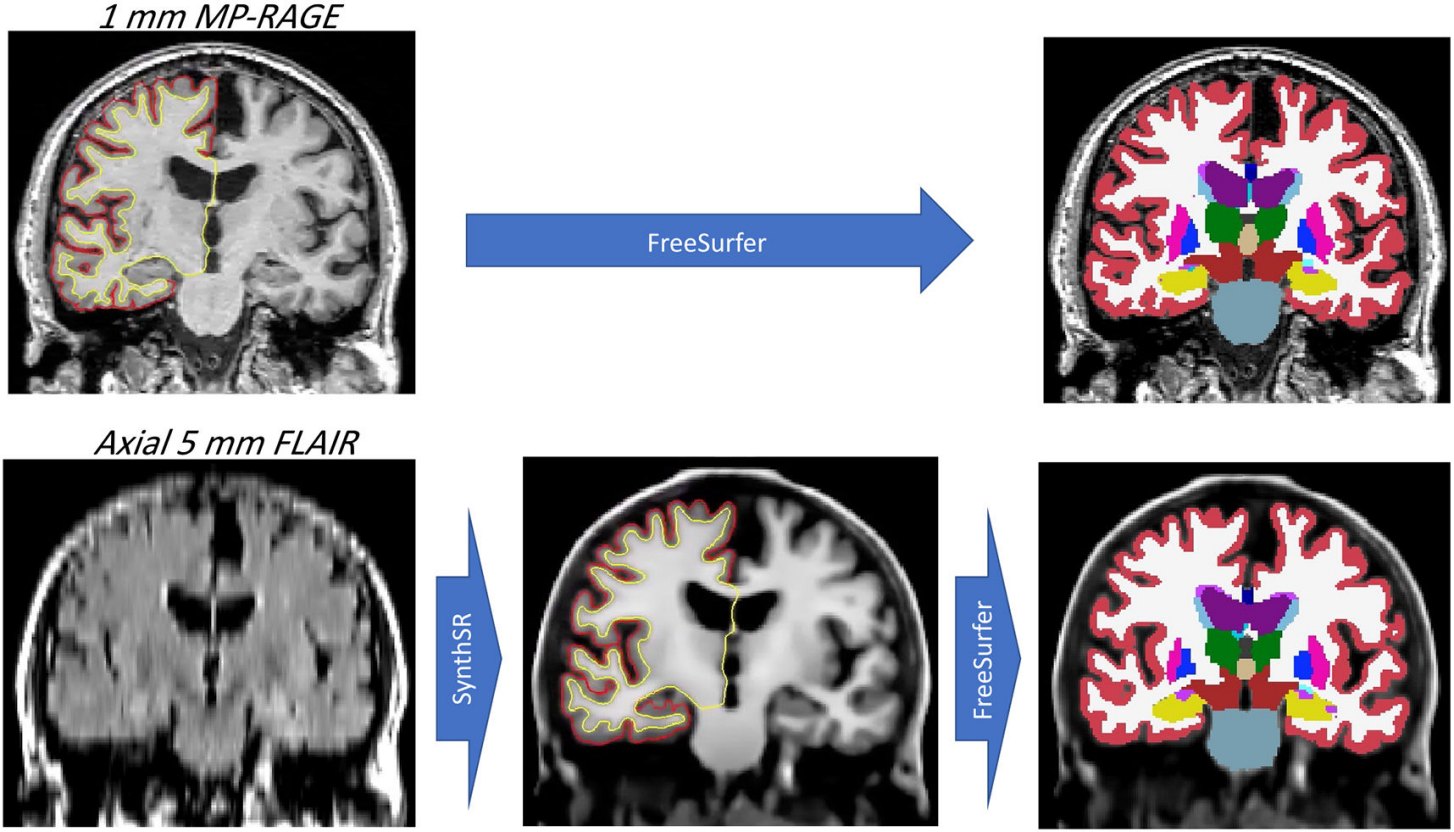
Left column: coronal plane of an MP-RAGE scan (top, slice thickness: 1 mm)) and corresponding coronal plane of an axial FLAIR scan (bottom, slice thickness: 5 mm) from the ADNI dataset (adni-info.org). Middle column: synthetic 1 mm MP-RAGE volume produced by Dr. Iglesias’s tool. Right column: automated segmentation of the original and synthetic MP-RAGE volumes produced by FreeSurfer (Fischl et al., 2002)

It is now possible to aggregate larger cohorts of useful brain image data from clinical and/or research archives by using deep learning to transform lowerquality images into higher-quality ones, thus enabling use of advanced image segmentation tools. In particular, several such tools are built for MP-RAGE scans, which are popular due to their SNR efficiency and contrast. At this symposium, Dr. Juan Eugenio Iglesias presented an approach to synthesize isotropic 1 mm MP-RAGE volumes from low-resolution scans of arbitrary contrast, enabling their segmentation and analysis with standard neuroimaging tools (Iglesias et al., 2020). An example is shown in Fig. 5, where a 5 mm axial FLAIR scan is transformed into a 1 mm isotropic MP-RAGE scan, and subsequently segmented with FreeSurfer, which requires 1 mm isotropic T1 data—and thus could not have processed the FLAIR scan directly, due to MR contrast mismatch and insufficient resolution.

### Visualization

Visualization frameworks can foster deeper understanding and facilitate interpretation of high-dimensional clinical imaging data. Further, targeted visualizations allow developers to design and optimize computational algorithms. State-of-the-art visualization tools deal with challenges such as large amounts of data such as in diffusion and functional MRI, and the inevitable variation of file formats across different institutions. Web-based tools such as Fiberweb (Ledoux et al., 2017) or XTK (Haehn et al., 2014) have contributed to brain imaging visualizations and 3D rendering of connectivity in recent years. Many other visualization tools not limited to DTI data emerged recently, such as Neurolines (Al-Awami et al., 2014) to visualize 3D brain tissue in 2D, and comparative visualizations for fMRI brain images (Jönsson et al., 2019).

**Fig. 6:**
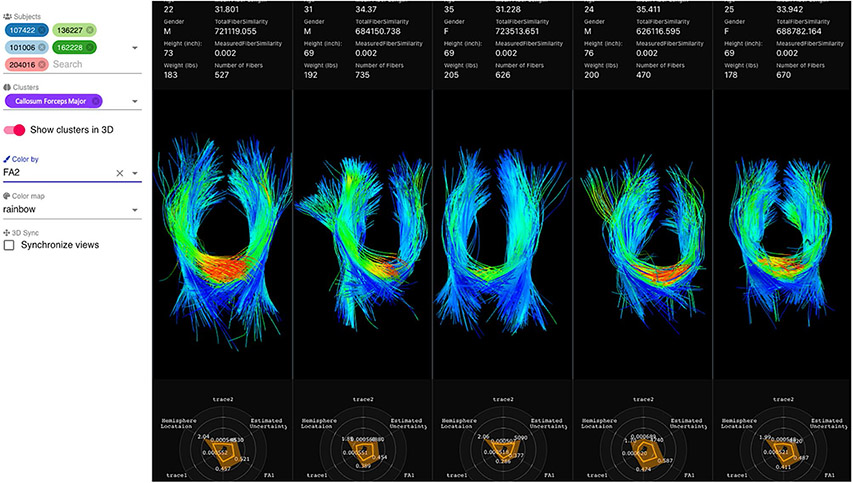
Split screen showing 3D representations of fiber tract anatomy given by fibers of dMRI scans across different subjects. The menu bar at the left facilitates toggling on and off visualization of different subjects (top left), cluster (middle left, showing the Callosum Forceps Major), and coloring of the 3D tract by a selected scalar value (bottom left, showing a measurement of fractional anisotropy (FA2) from the DTI scan). High values of fractional anisotropy are colored in red while lower values are colored in blue. Inter-hemisphere crossing of the third patient shows no red colors and therefore no high fractional anisotropy values. Tractography from five different patients is displayed with additional two-dimensional representation radial plots at the bottom of each patient’s panel showing scalar values associated with each of the fiber tracts. For each anatomical tract, the 2D radial plots show mean and standard deviations of the different scalars on each axis. Demographic information about each patient is shown above the 3D visualization, for example, age, gender, height and weight. Each patient is anonymized by a number seen in the labels next to the anatomical fiber tract name in purple. Other relevant measurements for analysis are mean fiber length, number of fibers or fiber similarity

At the symposium, Ms. Loraine Franke presented her work on developing web-based interactive visualization tools for diffusion tractography imaging data (Franke & Haehn, 2020; Franke et al., 2020). Her open-source tool, FiberStars (Franke et al., 2020) (Fig. 6) enables researchers to create low-dimensional cluster representations of high dimensional data, select, visualize, and compare multiple clusters across multiple patients, and visualize individual patient fiber tracts. By using different projection techniques for multidimensional scaling such as t-SNE (van der Maaten & Hinton, 2008), PivotMDS (Brandes & Pich, 2007) and others, the FiberStars tool lets the user interactively explore high dimensional data. For example, FiberStars enables users to answer research questions with comparative ensemble visualizations, especially for evaluating and testing hypotheses, or to analyze factors combined with pathological findings. FiberStars addresses a large class of complex visualization challenges for multidimensional data or data composed of collections of patients.

## Section III: Machine Learning Advances in Interpretation and Analysis of Non-volumetric EEG Data

Another important, and emerging, area where machine learning approaches are enhancing the understanding of brain health is in clinical applications of electroencephalography, or EEG data. EEG is multidimensional time series data, where multiple electrodes are placed on the scalp resulting in simultaneous channels of data being collected at a high time resolution. EEG is currently in clinical use for multiple, specific applications, such as for diagnosing and monitoring sleep disorders, epilepsy, disorders of consciousness, stroke, real-time electroconvulsive therapy (ECT) patient monitoring, and anesthesia (Roy et al., 2019a, b). EEG has the unique advantages of being non-invasive, relatively inexpensive, and more adaptable to naturalistic or ambulatory settings compared to other imaging modalities. In some cases, even a few EEG electrodes in a specific location can yield enough information for inference, without the need for EEG across all of the cortex. Therefore, machine learning approaches can not only greatly streamline existing clinical applications of EEG, but they can also open the door to new applications such as earlier, less expensive, or more accessible diagnostics (Michel & Murray, 2012; Miranda et al., 2019).

Some of the most advanced work focuses on the diagnostic needs for patients with epilepsy, an area for which EEG is already in active clinical use. In the current standard of care, making diagnoses and therapeutic decisions relies on painstaking manual annotation of many hours of EEG recording by highly-trained expert epileptologists (Si, 2020). Machine learning methods enable automatic detection of markers of epilepsy in interictal (non-seizure) data using specific spectral, morphological, or network-based features. While some feature-based approaches attempt to replicate the eye of the expert using features like those epileptologists observe; other end-to-end deep learning and neural network models attempt to glean undiscovered signatures of epilepsy from the raw data itself. One example of this is classifying routine EEGs into normal vs. abnormal, where abnormal is, by definition, heterogeneous and context-dependent (van Leeuwen et al., 2019). Machine learning based clinical decision support for epileptologists for diagnosis and localization of epileptic foci are highly promising as they reveal interrelationships between brain regions and activity that are difficult to discern by eye.

In contrast to epilepsy, where EEG is already being used clinically, machine learning approaches are expanding the potential for EEG-based diagnostic biomarkers for other diseases, such as Alzheimer’s Disease (Escudero et al., 2006; Gallego-Jutglà et al., 2015; Jelles et al., 1999; Lehmann et al., 2007; Tzimourta et al., 2021; Woon et al., 2007). However, these methods are further from clinical deployment than those for epilepsy, mostly in feature discovery stages. Analogous strides are being made to discover novel, cost-effective, and ambulatory EEG-based biomarkers for diagnosing stroke, schizophrenia, and attention deficit hyperactivity disorder (Ahmadlou & Adeli, 2011; Hosseini et al., 2020; Phang et al., 2020; Sastra Kusuina Wijaya et al., 2015). While these directions have great potential for impact if successful, since they are new clinical applications of EEG, their success depends on connecting sound and robust machine learning algorithm design to underlying physiology, which can prove elusive. Interpretability will likely also come into play, since clinicians must be convinced of the specific clinical utility of EEG for each new application.

A recurring theme in the development of machine learning methods that is the same for EEG, as it is for any other imaging modality, is the availability of large, labeled datasets. Three such sources for large EEG datasets are the National Sleep Research Resource (Sleep Data—National Sleep Research Resource, 2021 https://sleepdata.org/), the PhysioNet Computing in Cardiology Challenge 2018 (Ghassemi et al., 2018), and the TUH Abnormal EEG corpus (Alhussein et al., 2019; Gemein et al., 2020; Roy et al., 2019a, b; Temple University EEG Corpus Downloads, 2021).

## Section IV: Brain Health as Assessed by Deviations from Healthy Aging

**Fig. 7:**
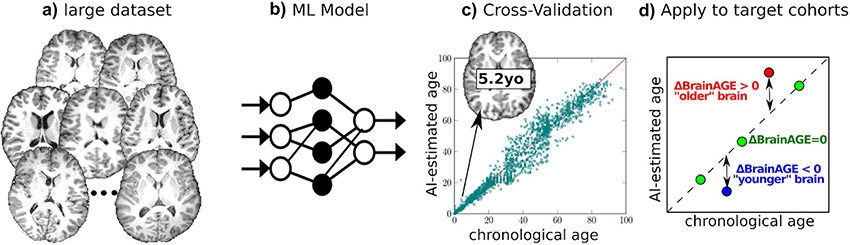
Machine learning (ML) can estimate a patient’s brain age and quantify abnormal (accelerated or delayed) aging. (**a**) training samples consisting of normal brain MRIs from a large set of individuals; (**b**) ML algorithm that learns how a normal brain MRI appears at various ages; (**c**) cross validation to quantify the accuracy of the ML model; and (**d**) when applied to target patients, the ML model can quantify deviations from normal brain aging

Another machine learning approach to characterize brain health is to summarize an image or biosignal into a single metric that reflects brain health, such as brain age estimation (Fig. 7d) (Cole et al., 2019). The difference between estimated brain age and actual chronologic age, known variously as predicted age difference (PAD), Brain Age Index (BAI) or ΔBrainAGE, has identified accelerated aging in individuals with cognitive impairment (Liem et al., 2017; Poddar et al., 2019), traumatic brain injuries (Cole et al., 2015), schizophrenia (Cole et al., 2018), Alzheimer's disease (Bashyam et al., 2020), and diabetes (Franke et al., 2013). Deviations from expected brain age have also been reported for more subtle changes due to social and environmental influences, including a protective decrease in brain aging for long-term meditation practice (Luders et al., 2016), music-making (Rogenmoser et al., 2018), and a higher level of education (Steffener et al., 2016), as well as accelerated aging associated with smoking and alcohol consumption (Guggenmos et al., 2017; Ning et al., 2020).

At the symposium, Dr. Ou presented his recent work (He et al., 2020, 2021) on a novel, deep convolutional neural network brain age prediction model that uses both morphological and contrast-based changes in brain MRI data to estimate brain age. This work was enabled by collating 11 different data sets and carefully curating a very large, harmonized dataset that included enough healthy subjects of all ages to train, test and validate the method (Fig. 7a). By explicitly splitting the T1-weighted brain MRI into morphometry (spatial information) and contrast (tissue based signal information) channels, his attention-driven multi-channel fusion network (Fig. 7b) improved the accuracy of age estimation as compared to each channel alone, or naive fusion of two channels without their proposed attention mechanisms, when applied to 16,705 normal brain MRIs acquired over the lifespan (0–97 years of age) (He et al., 2021). The team cross validated their work against multiple published brain age estimation algorithms and using multiple independent test data sets (Fig. 7c). A critical advantage of this end-to-end method is that it has the potential to differentiate between abnormal aging associated with contrast change (e.g., lesions) and those associated with morphometric changes (e.g., atrophy). This is an important contribution toward increasing the specificity of brain age estimator biomarkers, a major issue for this line of research (Kaufmann et al., 2019).

**Fig. 8:**
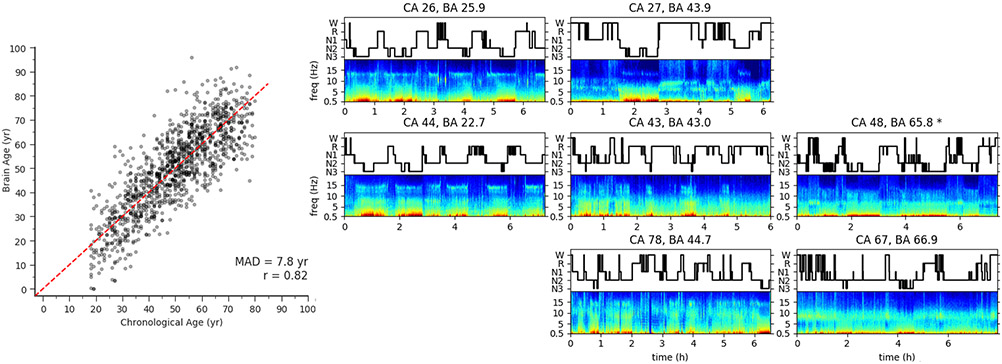
Illustration of sleep EEG-based brain age. (Left) The scatter plot of chronological age vs. brain age where the diagonal dashed red line indicates where chronological age equals brain age. The mean absolute deviation (MAD) is 7.8 years and Pearson’s correlation R = 0.82. (Right) The confusion matrix of example EEG spectrograms (bottom in each subplot) and hypnogram (trajectory of sleep stages) (top in each subplot), where the top, middle, and bottom rows are patients with young, middle, and old chronological age (CA, in years) respectively; while the left, middle, and right columns are subjects with young, middle, and old brain age (BA, in years). Comparison within each row reveals different sleep EEG microstructures for different brain ages while at similar chronological age

Another symposium speaker, Dr. Haoqi Sun, presented his work on a feature-based machine learning model that takes advantage of the fact that brain activity as recorded by EEG during sleep naturally varies with age (Leone et al., 2021; Paixao et al., 2020; Sun et al., 2019; Ye et al., 2020). Features from both time and frequency domains of each sleep stage are used to compute an overall brain age. Figure 8 shows the scatter plot of chronological age vs. sleep EEG-predicted brain age, and eight example sleep EEGs from across the lifespan with their chronological age and calculated brain age shown. Dr. Sun showed that across two large sleep EEG datasets, people with significant neurological or psychiatric disease show a mean excess brain age (compared to chronological age) of 4 years compared to healthy controls on a population level, while those with hypertension or diabetes show a mean excess brain age of 3.5 years compared to healthy controls (Sun et al., 2019). Sun and colleagues have validated the association of significant differences between sleep EEG based age and chronological age in patients with dementia and MCI (Ye et al., 2020), people diagnosed with HIV under antiretroviral therapy (Leone et al., 2021), and all cause mortality (Paixao et al., 2020).

As with sleep EEG, features of brain activity under general anesthesia have also been demonstrated to change with age, allowing the EEG patterns measured during administration of general anesthesia to be evaluated as a marker of brain age (Akeju et al., 2015; Lee et al., 2017; Purdon et al., 2015). Similar ideas about indicators of brain health under general anesthesia are motivating the development of EEG machine learning methods to monitor and assess disorders of consciousness, since no other behavioral markers can be used (Engemann et al., 2018).

A fundamental challenge in using brain age estimation as an index of brain health and/or meaningful clinical indicator is that the rate of age-related changes in brain structure and function (e.g. sleep) vary across the lifespan such that early and late life changes are more readily detected, but are very subtle between 30 and 60 years of age. For both MRI- and EEG-based brain age prediction, the sensitivity is lowest during this part of the lifespan. Not surprisingly, one promising application of MRI-based brain age prediction is early detection of future neuropsychiatric disorders in children and/or adolescents (Chung et al., 2018). The relative stability of structural MRI measures bound the temporal resolution of brain age estimates using that modality (Cole & Franke, 2017; Karch et al., 2019), while the significantly higher night-to-night variability of sleep EEG-based brain age estimates is both a challenge to overcome if looking for stability, but also a potential additional source of meaningful signal to exploit in future work (Arnal et al., 2020; Arnardottir et al., 2021; Hogan et al., 2021).

There are important caveats to the use of a brain age as a marker of brain health since deviations from chronological age could be due to multiple factors. Brain age estimation studies remain population-level statistical tests because current approaches lack the sensitivity to accurately assess clinically meaningful deviation at the individual patient level. Because of these limitations, brain age is currently viewed as a screening tool where large deviations call for further investigation. More work is required to improve the specificity and clinical utility of brain age estimation. Combining EEG- and MRI-based brain-age estimation techniques with and without additional features (e.g. genomic markers, demographics, socioeconomics status, and environmental factors) to more accurately predict disease status at individual level is an active area of research (Al Zoubi et al., 2018; Cole & Franke, 2017; Mohajer et al., 2020; Varikuti et al., 2018). For example, in the case of sleep EEG-based brain age, the density of sleep spindles (count/hour) one of the features used in the model, appears to be a heritable trait based on the expression of CACNA1l, a gene that is associated with both schizophrenia and sleep spindle formation (Merikanto et al., 2019).

Despite these limitations, it is intriguing, and potentially clinically advantageous, that lifestyle choices such as exercise and sleep can modify these quantitative metrics of brain age in directions that reflect known associations with brain health. Studies have shown that actively exercising leads to an orchestra of changes in energy metabolism, oxidative stress, inflammation, tissue repair, growth factor response, and regulatory pathways in the brain (Contrepois et al., 2020). Sleep has a bidirectional relationship with the immune system (Irwin, 2019), therefore there is evidence for and reason to expect that exercise can improve sleep and thereby improve brain health, which will be reflected in normalized sleep-based brain age biomarkers in people with evidence of accelerated aging.

## Section V: Application of Machine Learning Techniques for Diagnostics, Prognostication, and Personalization of Medical Treatments

Imaging plays a key role in the clinical evaluation of pathological changes that can be readily distinguished from a healthy brain. Neuroradiologists routinely use neuroimaging modalities such as CT, MRI, and PET for both qualitative and quantitative assessment of diseases from infectious, autoimmune, oncological, degenerative, and vascular etiologies. However, despite standardization efforts, manual assessment is subject to inter- and intrarater variability (Filippi et al., 1995; Provenzale & Mancini, 2012; Provenzale et al., 2009; van Horn et al., 2021). As such, there is intense interest in automating radiological assessment with machine learning. A popular approach is radiomics (Beig et al., 2020), which focuses on the extraction of pertinent quantitative imaging features often followed by incorporation of these features into a predictive machine learning algorithm. These imaging features are computational imaging descriptors reflecting measures such as size, shape, intensity distribution, and intensity heterogeneity (Zhou et al., 2018). Indeed, these feature-based radiomic approaches have found success for early detection (Sørensen et al., 2016), diagnosis (Kniep et al., 2019; Regenhardt et al., 2021; Tanioka et al., 2020; Zhou et al., 2020), prognostication (Macyszyn et al., 2016; Stefano et al., 2020; Tang et al., 2020), treatment response prediction/assessment (Cai et al., 2020; Chang et al., 2016; Hofmeister et al., 2020), and non-invasive determination of molecular markers (Beig et al., 2018; Pan et al., 2019) for a wide variety of diseases. More recently, deep learning approaches (Chang et al., 2018a, b; Rauschecker et al., 2020; Titano et al., 2018) have gained traction for similar tasks due to these approaches foregoing the need to pre-engineer imaging features. Some approaches have even shown the utility of combining radiomics with deep learning (Lao et al., 2017; Xiao et al., 2019). While there is great promise for these automated approaches, they do not come without pitfalls. Radiomics, in particular, has been challenged by variability stemming from differences in image acquisition, pre-processing, segmentation, and feature implementation (Hoebel et al., 2021; Kalpathy-Cramer et al., 2016; Schwier et al., 2019). Approaches to rectify these sources of variability and harmonize radiomic features have been an active area of study (Carré et al., 2020; Marcadent et al., 2020; Orlhac et al., 2018; Parmar et al., 2014; Zwanenburg et al., 2020). Similarly, deep learning approaches also suffer from a lack of generalizability across different image acquisition settings and patient populations (AlBadawy et al., 2018; Chang et al., 2020; Zech et al., 2018). These challenges will need to be addressed before these automated approaches can be effectively utilized.

Beyond its neuroradiologic applications to promote brain health with early detection, diagnostics, or prognostication related to neurologic disease, machine learning also has applications towards brain health as it relates to precision medicine—i.e. the development of personalized interventional therapies for a broader range of neuropsychiatric disorders informed by both data from a specific patient and aggregated information from larger patient datasets (Calhoun et al., 2021; Vieira et al., 2017; Zhang et al., 2020).

Data-driven precision therapeutics are already being translated to the clinic using transcranial magnetic stimulation (TMS) and other targeted brain stimulation approaches. At the symposium, Dr. Shan Siddiqi presented on this work, highlighting that TMS targets for any given symptom may be identified based on the location of brain lesions that cause the same symptom (Cash et al., 2020; Davey & Riehl, 2005).

Complementing Dr. Siddiqi and his team’s research is a large body of work focused on machine learning-based target optimization of field distributions for transcranial magnetic and/or electric stimulation that factor in the biophysical properties of biological tissues or feedback from real-time fMRI. “The Automatic Neuroscientist” framework uses real-time fMRI in combination with Bayesian optimization “to automatically design the optimal experiment to evoke a desired target brain state.” (Lorenz et al., 2016). Machine learning techniques have been applied towards rt-fMRI neurofeedback studies, where a neurofeedback signal can be derived using supervised learning methods such as linear models and support vector machines (LaConte et al., 2007).

In addition, both data driven and hypothesis driven analyses of functional connectivity data have been used to predict clinical outcomes including treatment response in patients (Whitfield-Gabrieli et al., 2016) as well as to predict pediatric vulnerability to psychiatric disorders including psychosis (Collin et al., 2019, 2020), depression (Chai et al., 2015), anxiety, and ADHD (Collin et al., 2020; Cui et al., 2020). At the symposium, Dr. Susan Whitfield-Gabrieli presented on these approaches, sharing evidence that connectivity between the medial prefrontal cortex (MPFC) and the dorsolateral prefrontal cortex (DLFPC) can be used as a biomarker to predict attentional problems in a normative pediatric population as assessed four years later, where greater baseline MPFC-DLPFC connectivity predicted worsening of attentional issues (Whitfield-Gabrieli et al., 2020) while decreased baseline subgenual anterior cingulate (sgACC)—DLPFC connectivity predicted worsening of anxiety/depression. As psychiatric neuroimaging research has evolved from the description of patient cohorts using simple group comparisons towards a focus on *individual differences* and “predictive” analytics, preliminary studies suggest that *intra-individual* fluctuations of brain activity provide better prediction of symptoms than group-based studies. Machine learning integrated with experience-sampling can be used to produce novel brain-based predictive models of state fluctuations (e.g., fluctuations of mind wandering) which generalizes to both healthy and clinical populations (Kucyi et al., 2021). Dr. Whitfield-Gabrieli also highlighted the use of mindfulness based rt-fMRI neurofeedback as a non-invasive, personalized circuit therapeutic to reduce symptom severity in psychotic patients as well as for teens with major depressive disorder and/or anxiety. (Bauer et al., 2020; Stoeckel et al., 2014) These pioneering studies provide strong motivation to pursue imaging based treatments.

Overall, machine learning based methods have potential to augment diagnostic and treatment workflows. As with all clinical interventions, the overarching goal is to improve patient outcomes, either within a specific decision point or longitudinally. While promising, more rigorous prospective and external validation studies in diverse clinical scenarios and populations are needed before these methods can be deployed for widespread use.

## Section VI: Additional Considerations for Clinical Deployment

### Regulatory Framework

To deploy any of the advances highlighted in the symposium that use machine learning algorithms in clinical practice, proposals must first clear the regulatory process as set by the Center for Devices and Radiological Health within the FDA that handles medical devices. Most machine learning methods in healthcare are categorized as *software as a medical device* (SaMD) which is a subcategory under *software related to medical devices* under the *medical device* umbrella. The pathway to market depends on the risk associated with the software, which in turn depends primarily on 1) significance of information provided by SaMD to a healthcare decision and 2) state of healthcare situation or condition; a more critical situation yields a higher risk rating. Traditionally, SaMD algorithms need to be locked, i.e., give the same output for the same input, after they are submitted to the FDA for premarket approval. This is impractical for machine learning software in situations where it is often desirable to continuously update the machine learning models based on user data (e.g. to accommodate updates in scanner hardware and software). The FDA has proposed a new regulatory framework based on a total product life cycle approach, wherein the initial premarket submission outlines the modifications that might take place in the future. The manufacturer can then continuously update their machine learning models based on new user data without having to go through a new premarket submission provided that the update is within the SaMD Pre-Specifications and algorithm change protocol (Digital Health Center of Excellence, 2021, https://www.fda.gov/medical-devices/digital-health-center-excellence/software-medical-device-samd). These guidelines are under active discussion, development, and refinement in collaboration with industry, academic, and clinical leaders.

Machine intelligence in medical imaging is one of the most vibrant fields within the application of machine learning in healthcare, and one of its biggest subfields is quantitative imaging (QI). QI refers to extraction of quantifiable features from medical images that serve as biomarkers for specific physiological conditions, such as features relating to aspects of brain health which have been discussed above. A premarket submission for a QI function requires a function description including the level of automation (manual, semi-automatic or fully automatic), a brief description of the training algorithm, quantitative performance specifications, and instructions used for semi-automatic labeling of the training set. The biggest part of the premarket submission is the technical performance assessment which should include a definition of the QI function, its relationship to the measurand, and the use conditions. For example, this could be a “brain age” assessment from MRI data applicable to images of a specific resolution collected on a specific MRI system. It should also specify the performance metrics and characterize the performance of the QI function under the predefined conditions. In the mentioned example, performance metrics could include accuracy as measured in deviation between actual age and estimated age in a normative cohort as well as bias or precision as measured in reproducibility or repeatability. A priori acceptance criteria regarding these performance metrics should also be set along with restrictions and limitations on usage, and the results of a study presented where the outcomes are compared to the predefined acceptance criteria.

### Ethical Considerations During Machine Learning Model Development

As the machine learning applications in this report mature in their development, there are a number of vital ethical issues to be taken into consideration. While not the primary focus of the symposium, both the organizer, Dr. Randy Gollub and keynote speaker, Dr. Simon Eickhoff emphasized the importance of these aspects, pointing out a few examples of how, where, and why they are relevant. Some of these ethical considerations have established guidelines or technical best practices that need to be more widely used; others are ongoing discussions for which there is not yet a clear-cut solution (see for example the Fair ML for Health Workshop that was held during the NeurIPS 2019 Workshop (Fair ML for Health—Accepted Papers, 2021, https://www.fairmlforhealth.com/accepted-papers). It is crucial that scientists and researchers participate actively in these discussions at each stage of development of these methods. We note that this section of our report is by no means comprehensive; for more in-depth discussions of these issues, see (Beauvais et al., 2021) and (Chen et al., 2020).

#### Data Sharing

Data sharing across institutions may eventually become necessary to create large enough datasets to train sophisticated machine learning algorithms. To mitigate the risks of breach of privacy, security, and confidentiality, robust de-identification algorithms that retain all necessary imaging data elements are essential, and all modalities of data must be scrutinized to ensure that there are not additional unintended sources of protected information amongst them (e.g. private DICOM metadata tags). Secure cloud servers and backup protocols, expert curation and maintenance, and strict guidelines and training for researchers on how to securely access, store, and dispose of data are all additional tools to minimize the chances of confidentiality breaches or loss of data. Federated learning methods which allow data to be stored only at the location where it was collected while allowing for multisite analysis are another means to support robust, yet protected, data sharing (K. Chang, Balachandar, et al., 2018; Chang, Grinband, et al., 2018). For all these approaches, frequent communication between all institutions involved will also ensure that everyone is kept apprised of possible issues in a timely manner and that any changes are implemented in an organized and efficient manner.

#### Informed Consent for Expanded or Later Use of Data

It is becoming increasingly common for large datasets to be used and reused in multiple studies and towards different machine learning algorithms once they have been collected. This is mainly due to the cost in time, money, and resources to amass an entirely new dataset for each research question. Repurposing existing datasets across many studies is overall a very efficient and effective option; however, the wishes of those from whom the data is collected must be respected. Most current informed consent paradigms are based on data being collected for a single study and therefore obtain informed consent from a patient for that single study alone. However, this system needs to be modified to reflect that it is likely a patient’s data could be used for many studies even decades into the future, most of which cannot even be fathomed at the time of data collection. Patients should, at minimum, have the ability to ‘opt out’ of having their data used in future studies without their explicit consent. Many current guidelines state that if the data is de-identified, it can be shared and used for new studies without re-obtaining consent, often after obtaining a waiver of consent from the local Institutional Review Board. However, current trends warrant reexamination of these guidelines with a goal of securing patient consent for wider, protected data use at the time of enrollment.

#### Intellectual Property and Commercialization

Clearer intellectual property guidelines are needed regarding models or algorithms developed from patient data collected for research use. With the increasing use of large datasets for multiple studies and across long periods of time, it is difficult to track all of the downstream uses of a single person’s data. After data collection, development and validation of new methods, these methods may eventually be commercialized. However, inherent to any trained machine learning model or algorithm is the data that was used for such training. The data is inextricably tied to any intellectual property or commercial potential that results from the development process. Is it fair to allow patenting of trained models or algorithms on data collected from people who did not consent to its possible use for commercial profit? Should those people be included in any such profit? These questions must be answered as machine learning models become integrated into clinical pipelines.

#### Bias in Datasets

In machine learning, the phrase “garbage in, garbage out” reflects the fact that bias, noise, or flaws in the underlying data used to train a model will undoubtedly affect the quality, accuracy, and validity of the results. Therefore, ensuring high quality data that is highly representative of the populations under study is paramount to the ethical and effective development of these methods. One key component of this for assessments of brain health is ensuring adequate representation of traditionally underrepresented subpopulations in research, including underrepresented minorities, women, low and middle income nationals, transgender and gender non-conforming individuals, undocumented immigrants, and pregnant women, especially from an intersectional lens. This is especially important because of the specific mental and behavioral health issues which impact brain health in many of these subpopulations. It also includes considerations in the study design itself to ensure these populations are not excluded inadvertently by data acquisition methods, for example by failing to include more than the traditional binary options when documenting gender. Even if the intentions of researchers are to include all populations, other aspects of the study design can inadvertently be biased towards certain populations. For example, studies that require mobile phone downloads of certain apps or track social media use exclude populations who do not have access to smartphones or social media.

Even once a study is underway, oversight and periodic assessments of study recruitment practices should be done to check for inadvertent exclusion of certain populations. For example, the inclusion and exclusion criteria of many studies, especially randomized controlled trials, are often written with purely scientific or clinical considerations in mind relating to the treatment or diagnostic in question. However, they can result in a study population that is too restricted and not reflective of the actual population of interest. Studies that involve multiple study visits at different times may discourage participation of those who do not have the access or flexibility to come to the research site several times. Any researcher using existing datasets should hold to these same standards when checking the subject profile of the already collected data and include this information, including limitations of the dataset, in any resulting publications.

Technical approaches have been suggested as tools for handling disproportionate representation of certain subgroups within large datasets. For example, some approaches force neural networks to learn intermediate representations which cannot be used to predict a protected attribute of interest, e.g. gender or race (Dinsdale et al., 2021).

#### Quantifying/understanding Uncertainty

Whenever possible, researchers and scientists should attempt to quantify the uncertainty of their model predictions using statistical tools such as the confidence interval. Before such algorithms are implemented, clinicians should receive training on how to interpret the results given the limitations of any model, including uncertainty. There is much attention being focused on these issues, including annual workshops that have been held since 2019 at the MICCAI meetings- “UNSURE Uncertainty for Safe Utilization of Machine Learning in Medical Imaging” with presentations and awards for work in areas such as risk management of machine learning systems in clinical pipelines, measurement errors, methods for modeling noise in data, validation of uncertainty estimates, calibration of uncertainty measures and more (Greenspan et al., 2019).

### Ethical Considerations at the Stage of Deploying a Machine Learning Model

#### Privacy and Confidentiality of Information

As machine learning tools for brain health emerge, an important question to answer is: who should have access to the outputs of these methods? Should the patient have unfettered access to their own ‘brain health’ information? Should all clinicians who might interact with that patient have access? If it will be integrated into the medical record, how do we prevent it from affecting billing or insurance practices? How might the information bias someone’s interaction with a patient, especially without accurate reporting (with uncertainty)? These are all considerations that cannot be taken lightly and will have to be addressed to develop clinical best practices.

#### Incidental Findings

As with genetic information and testing, the probabilities of incidental findings in large datasets such as neuroimaging are non-trivial. How should such findings be handled? Do clinicians have an obligation to inform patients? What about for measures such as one of the indices of ‘brain age’ for which the implications are still under study? Clear clinical practice guidelines for the handling of sensitive information relating to brain health must be developed prior to the deployment of any such algorithm.

While many of these ethical considerations are gray areas for which we can only postulate guidelines and not clear answers, ongoing discussion will hopefully lead to new best practices that adhere to the highest ethical standards on each of the issues discussed, to safeguard patients and their anatomic and physiological datasets. For researchers, it is important to keep in mind that the misinterpretation or generalization of one poorly designed high-profile study or one breach in confidentiality can be enough for an entire field to lose credibility.

## Conclusion

The MGH McCance Center for Brain Health and Harvard-MIT Health Sciences and Technology Neuroimaging Training Program co-hosted virtual symposium, “Neuroimaging Indicators of Brain Structure and Function—Closing the Gap Between Research and Clinical Application,” explored the recent explosion of machine learning approaches augmenting the clinical and scientific neuroimaging pipeline. Researchers presented cutting-edge techniques for acquiring more informative imaging data, more effectively analyzing this acquired data, and more precisely acting on the insights from this analysis to guide and individualize treatment decisions. The work presented at this symposium highlighted several open research directions which must be explored in order to implement these techniques in practice. For example, progress in this field will require techniques for robust generalization of machine learning techniques to more realistic, heterogeneous datasets as well as methods for identifying the uncertainty present in machine learning-based predictions and presenting this information to end users within a clinical workflow. Toward these ends, our field will need to ensure the availability of sufficiently large, curated data sets; the ability to share valuable data sets thus engaging a diverse, committed scientific community (Eickhoff et al., 2016); and responsible stewardship of brain imaging data to ensure appropriate protections for individual privacy as well as intellectual property and proper handling of bias in these data. With a firm commitment in these directions, machine learning promises to dramatically improve the early detection, prediction, and treatment of diseases that threaten brain health.

Note: Interested readers may view recorded videos of all symposium presentations and discussions at this link https://www.youtube.com/playlist?list=PL0A-NKHLVrNF82vdjeyyaBRoiXg77lCeW.
